# Bleached Kraft Eucalyptus Fibers as Reinforcement of Poly(Lactic Acid) for the Development of High-Performance Biocomposites

**DOI:** 10.3390/polym10070699

**Published:** 2018-06-24

**Authors:** Marc Delgado-Aguilar, Rafel Reixach, Quim Tarrés, Francesc X. Espinach, Pere Mutjé, José A. Méndez

**Affiliations:** 1 LEPAMAP Research Group, Department of Chemical Engineering, University of Girona, 17004 Girona, Spain; m.delgado@udg.edu (M.D.-A); joaquimagusti.tarres@udg.edu (Q.T.); pere.mutje@udg.edu (P.M.); jalberto.mendez@udg.edu (J.A.M.); 2 Department of Architecture and Construction Engineering, University of Girona, 17004 Girona, Spain; rafel.reixach@udg.edu; 3 Design, Development and Product Innovation, Department of Organization, Business Management and Product Design, University of Girona, 17004 Girona, Spain

**Keywords:** natural fibers, green composites, micro-mechanics, Kelly-Tyson, interphase

## Abstract

Poly(lactic acid) (PLA) is one of the most well-known biopolymers. PLA is bio-based, biocompatible, biodegradable, and easy to produce. This polymer has been used to create natural fiber reinforced composites. However, to produce high-performance and presumably biodegradable composites, the interphase between PLA and natural fibers still requires further study. As such, we aimed to produce PLA-based composites reinforced with a commercial bleached kraft eucalyptus pulp. To become a real alternative, fully biodegradable composites must have similar properties to commercial materials. The results found in this research support the competence of wood fiber reinforced PLA composites to replace other glass fiber reinforced polypropylene composites from a tensile property point of view. Furthermore, the micromechanics analysis showed that obtaining strong interphases between the PLA and the reinforcement is possible without using any coupling agent. This work shows the ability of totally bio-based composites that fulfill the principles of green chemistry to replace composites based on polyolefin and high contents of glass fiber. To the best knowledge of the authors, previous studies obtaining such properties or lower ones involved the use of reagents or the modification of the fiber surfaces.

## 1. Introduction

A wide variety of biodegradable and bio-based polymers are available, including thermoplastic starch (TPS), polyhydroxybutyrate (PHB), thermoplastic lignin, and polycaprolactones [1,2,3,4,5]. However, biopolymers still have some drawbacks in terms of costs and properties. Although producers have bargaining power due to low demand, the introduction of such biopolymers into the technosphere will be a challenging task. Fortunately, environmental awareness is increasing the demand and more products based on these biopolymers are being produced and introduced. 

**Figure 1:**
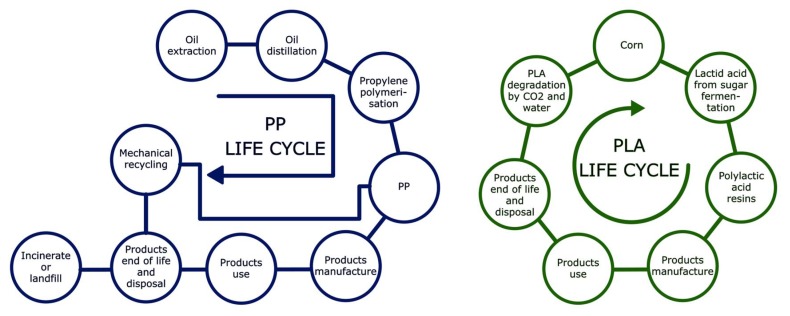
Simplified life cycle diagrams of polypropylene (PP) and poly(lactic acid) (PLA) resins used to manufacture consumer products.

Poly(lactic acid) (PLA) is becoming one of the most promising biopolymers in several fields, mainly due to its good mechanical properties, biocompatibility, and biodegradability. Although the potential applications of PLA are different than those of other commodities such as polypropylene (PP), polyethylene (PE), or even poly(vinyl chloride) (PVC) and poly(ethylene terephthalate) (PET). Previous works demonstrated that the use of properly modified, natural fibers increased the mechanical properties of PLA-based composites up to levels that made these materials competitive with the abovementioned materials [6]. The environmental advantage of PLA compared to other commodities is clear and has been extensively discussed [7,8,9]. Figure 1 shows the main difference, in terms of lifecycle, between PLA and PP, which was selected as the most representative oil-based commodity. 

Thermoplastic polymers are usually combined with fibers to further improve their properties. In fact, PP-based composites are mostly reinforced with glass fibers (GF), preventing their reuse and recycling, thus limiting their end of life to landfilling or incineration to recover some part of the material in the form of energy [10]. The substitution of these mineral fibers has become an interesting research topic, mainly due to increasing environmental awareness, safety issues, and governmental regulations [11]. The use of glass fibers has been reported to be harmful for thermoplastic processing equipment, such as injection molding machines or extruders, mainly due to their rigidity, hardness, and abrasiveness. Potential sustainable candidates as substitutes for these fibers have included natural fibers, either coming from wood [12], annual plants [13], or even recovered paper [14].

PLA has also been studied as a matrix in thermoplastic composites. However, as discussed in a previous study, the interphase between PLA and natural fibers requires further research. Although some evidence supports the presence of OH groups on fibers’ surface promoting the interaction between the polymer and reinforcement. Nonetheless, the resulting mechanical properties are still far from the market requirements, especially when compared to PP/GF composites [12]. Furthermore, like polyolefin, where removing lignin form the fibers surfaces has shown beneficial in order to enhance the mechanical properties of natural fiber reinforced composites, some authors found that lignin increased the interactions between PLA and the natural fiber surfaces [7,15]. This is of interest for the present research as supports the use of untreated natural fibers as PLA reinforcement. There are also other authors that propose mechanisms to increase the interactions between the phases of the composite, but involve the use of expensive coupling agents like montmorillonite or chemical modification, difficultly scalable to industry level, like acetylation [16].

Granda et al. (2016) used bleached kraft pine fibers as a PLA reinforcement, obtaining significant improvements when 30 wt % of fiber was used [6]. The tensile strength of PLA increased from 49.85 to 68.80 MPa, a value in the same magnitude of PP/GF composites with a 20 wt % content of reinforcement. Nonetheless, when the amount of GF increased to 30 wt %, a tensile strength of about 80 MPa was achieved. To the date, this value is not attainable with injection molding PLA grades and natural fibers. 

Given the above, the present work aimed to produce PLA composites reinforced with bleached kraft eucalyptus fibers, without any chemical modification or the use of any cross-linking agent, with comparable properties to PP/GF composites with the purpose of creating competitive and presumably biodegradable high-performance materials. The macromechanic and micromechanic properties, and interphase were studied to determine the influence of fiber morphology and composition. The results proved that it is possible to formulate and obtain totally bio-based composite materials with mechanical properties comparable to glass fiber reinforces polypropylene composites. The strength of the obtained materials was only 94% lower than a PP-based composite with 30% glass fiber content. To the best knowledge of the authors, this is the first time that these values have been obtained for a totally bio-based composite material, without the use of further reagents of coupling agents. 

## 2. Materials and Methods 

### 2.1. Materials

A PLA Ingeos Biopolymer 3251D, PLA-based polymer, by Nature Works (Blair, NE, USA) was used as biodegradable matrix for the composites. This thermoplastic has a volumetric index of 30 cc/10 min at 190 °C/2.1 kg. The bleached kraft hardwood fibers (BKHF) from eucalyptus were provided by LECTA, SA (Madrid, Spain). Diethylene glycol dimethyl ether (Diglyme), with a molecular weight of 134.17 g/mol, and a boiling point of 162 °C, provided by Clariant (Tarragona, Spain), was used as a dispersing agent during the composite compounding. 

All the chemical reagents used for fibers characterization, extraction, and bleaching were supplied by Scharlau, S.L. (Sentmenat, Spain).

### 2.2. Composite and Sample Preparation

The BKHF were pulped using an aqueous solution of 2/3 of Diglyme to avoid the formation of hydrogen bonds between cellulose fibers. These hydrogen bonds usually form during pulping and compounding. After pulping, fibers were dried at 105 °C until constant weight and then shred in a knife mill.

The composites were mixed in a Gelimat kinetic mixer (model G5S, Draiswerke, Mahwah, NJ, USA). The matrix and the reinforcement were introduced in the mixer at 300 rpm, then the speed was increased to 2500 rpm for 2 min, reaching a discharge temperature of 195 °C. Composites with 10, 20, and 30% *w/w* of BKHF were produced.

Composites were pelletized in a knife miller. The pellets were stored in an oven at 80 °C until needed in order to prevent moisture absorption.

Composite materials were mold injected using a Meteor 40 injection-machine by Mateu & Solé (Barcelona, Spain) to obtain standard ISO 527-1:2000 specimens. The pressure during mold filling was 120 kgf/cm^2^; afterward, a 37.5 kgf/cm^2^ holding pressure was applied. The machine has three heating areas that were tuned to 175, 175, and 190 °C. To ensure at least five specimens were available for the tensile tests, a minimum of 10 specimens for each composite were created. 

### 2.3. Mechanical Characterization

The methodology to tensile test the specimens agreed with the ISO 527-1:200 standard specifications. The specimens were stored in a Dycometal conditioning chamber at 23 °C and 50% relative humidity for 48 h prior to the mechanical testing. Then, at least 10 samples were tested to obtain the tensile strength, strain at break, and Young’s modulus of the composites. The tests were performed in a dynamometer DTC-10 supplied by IDMtest (San Sebastian, Spain), fitted with a 5 kN load cell and operating at a rate of 2 mm/min. For the evaluation of the Young’s modulus, a MFA 2 extensometer, by MF Mess and Feienwerktechnik GMBH (Velbert, Germany), was used to more precisely measure the deformation.

### 2.4. Fiber Extraction from the Composites

The reinforcing fibers were extracted from the composite by using a Soxhlet apparatus to dissolve the matrix. Small pieces of composite obtained from the mold-injected specimens were placed into the Soxhelt apparatus. Decalin vapors were refluxed for 24 h until the PLA matrix was completely dissolved. Then, the fibers were extracted from the apparatus and washed with acetone and water to remove all the remaining residues. Finally, the fibers were kept in an oven at 105 °C for 24 h to obtain dried fibers.

### 2.5. Morphologic Analysis of the Fibers

The length and width distributions of the reinforcement were measured with a MorFi Compact (morphological fiber analyzer) from Techpap SAS (Gières, France), following the ISO/FDIS 160652 standard. The equipment measured between 25,000 and 30,000 fibers. Four samples of each type of fiber were analyzed. The equipment returns the arithmetic (*l_a_*) and weighted (*l_l_*) mean lengths, computed as

(1) $$l_{a}=\frac{\sum_{i}n_{i}\cdot l_{i}}{\sum_{i}n_{i}};\;l_{l}=\frac{\sum_{i}n_{i}\cdot l_{i}^{2}}{\sum_{i}n_{i}\cdot l_{i}}$$

In some cases, if the amount of long fibers is scarce, a double weighted mean length (*l_w_*) is used with the micromechanics models. This double weighted length solves the statistic misrepresentation of the impact of the long fibers on the mechanical properties of the composite materials. 

## 3. Micromechanics

The micromechanics study encompassed the impact of the phase’s contents on the properties of a composite material. In the present work, the micromechanics of the Young’s modulus and the tensile strength were examined and calculated. The intrinsic properties of the reinforcements can be obtained experimentally, but this is difficult in some cases due to the morphology of the fibers. However, some authors defend that the intrinsic properties of a fiber inside and outside a composite can vary considerably [17,18]. In such cases, the literature recommends the use of micromechanics models, mainly in situations with good interphases between the reinforcement and the matrix [19,20,21,22]. 

### 3.1. Hirsch’s Model

Hirsch’s model combines the parallel and perpendicular models to equalize the orientation effects of the fibers on the Young’s modulus of the composite [23,24]

(2) $$E_{t}^{C}=\beta \cdot \left(E_{t}^{F}\cdot V^{F}+E_{t}^{M}\cdot \left(1-V^{F}\right)\right)+\left(1-\beta \right)\frac{E_{t}^{F}\cdot E_{t}^{M}}{E_{t}^{M}\cdot V^{F}+E_{t}^{F}\cdot \left(1-V^{F}\right)}$$

where *E_t_^C^, E_t_^F^*, and *E_t_^M^* are the Young’s modulus of the composite, the reinforcement, and the matrix, respectively. *V^F^* is the reinforcement volume fraction, and the factor *β* equalizes the parallel and perpendicular models. For natural fiber composites, a value of *β* = 0.4 has been reported to adequately reproduce the results obtained experimentally [25,26]. Once the Young’s modulus of the composite and matrix are experimentally obtained, it is possible to solve the equation to obtain the intrinsic Young’s modulus.

### 3.2. Modified Rule of Mixtures for the Young’s Modulus

Although some formulations have been introduced for a rule of mixtures for the Young’s modulus of short fiber semi-aligned reinforced composites, one of the most used is

(3) $$E_{t}^{C}=\eta_{l}\cdot \eta_{o}\cdot E_{t}^{F}\cdot V^{F}+\left(1-V^{F}\right)\cdot E_{t}^{M}$$

where *E_t_^C^*, *E_t_^M^*, and *E_t_^F^* are the Young’s modulus of the composite, the matrix, and the reinforcement, respectively; and *η_l_* and *η_o_* are the modulus length and orientation efficiency factors, respectively, used to equalize the contribution of the semi-aligned short reinforcement fibers. The modulus efficiency factor *η_e_* is obtained by multiplying the abovementioned efficiency factors (*η_e_* = *η_l_·η_o_*). In the equation, the term *η_e_*·*E_t_^F^* is referred to as the neat contribution of the fibers to the Young’s modulus of the composite [24,27,28]. 

Once the intrinsic Young’s modulus of the reinforcement is obtained the Hirsch model, Equation (3) can be used to compute the value of *η_e_*. The modulus efficiency factor is a measure of the stiffening yield of the reinforcement.

### 3.3. Cox and Krenchel’s Model

The main factors impacting the efficiency of the reinforcement contribution are the morphology and the orientation of the fibers. To evaluate the impact of the morphology, the Cox and Krenchel’s model [29,30] was used

(4) $$\eta_{l}=1-\frac{tanh\left(\beta \cdot l^{F}/2\right)}{\left(\beta \cdot l^{F}/2\right)}$$

with

(5) $$\beta =\frac{1}{r^{F}}\sqrt{\frac{E_{t}^{M}}{E_{t}^{F}\left(1-\upsilon \right)\cdot Ln\sqrt{\pi /4\cdot V^{F}}}}$$

where *β* is a coefficient for the stress concentration rate at the ends of the fibers, *r^F^* is the mean fiber radius, *l^F^* is the fiber’s weighted length, and *ν* is the Poisson’s ratio of the matrix (0.42 for PLA) [31]. The efficiency factor *η_e_* can be expressed as *η_e_* = *η_o_·η*_l_ and the identity was used to calculate *η_o_*. 

### 3.4. Modified Rule of Mixtures for the Tensile Strength 

Like the Young’s modulus, some formulations exist for the rule of mixtures for the tensile strength of a composite. With short semi-aligned fiber reinforced composites, the literature has introduced a set of modifiers to equalize the contribution of the reinforcement to the tensile strength of the composite. The main parameters impacting this contribution are the morphology of the reinforcement, its orientation relative to the loads, its grade of dispersion, the chemical nature of the phases, its mechanical properties, and its content [27,32,33]. An accepted formulation of this modified rule of mixtures (mRoM) for the tensile strength is

(6) $$\sigma_{t}^{C}\;=\chi_{1}\cdot \chi_{2}\cdot \sigma_{t}^{F}\cdot V^{F}+\left(1-V^{F}\right)\cdot \sigma_{t}^{M*}$$

where *σ_t_^X^* refers to the tensile strength of *X*. *X* can be the composite (*C*), the reinforcement (*F*), or the matrix (*M*). The asterisk after *M* indicates that the tensile strength of the matrix is not its ultimate value but the contribution of the matrix to the strength of the composite or the corresponding stress of the matrix at the strain at break of the composite. The composite is reinforced with semi-aligned short fibers and the model adds the parameters *χ*_1_ and *χ*_2_ as the orientation and the length factors, respectively. A more general model presents both parameters as a coupling factor *f_c_* (*fc = χ*_1_*·χ*_2_). The literature shows that composites with strong interphases have coupling factors in the range of 0.18 to 0.2 [12,34]. The term *fc·σ_t_^F^* has been defined by some authors as the neat contribution of the reinforcement to composite’s strength [18,32].

Similarly to the Young’s modulus, the tensile strength of the composites and the matrix, but not the intrinsic strength of the fibers, can be obtained experimentally. As mentioned above, some authors found noticeable differences between the intrinsic values obtained experimentally and the back-calculated values [17]. We propose the use of micromechanics models, validated by the literature, to obtain the intrinsic tensile strength of the reinforcement.

### 3.5. Modified Kelly and Tyson Equation

**Figure 2:**
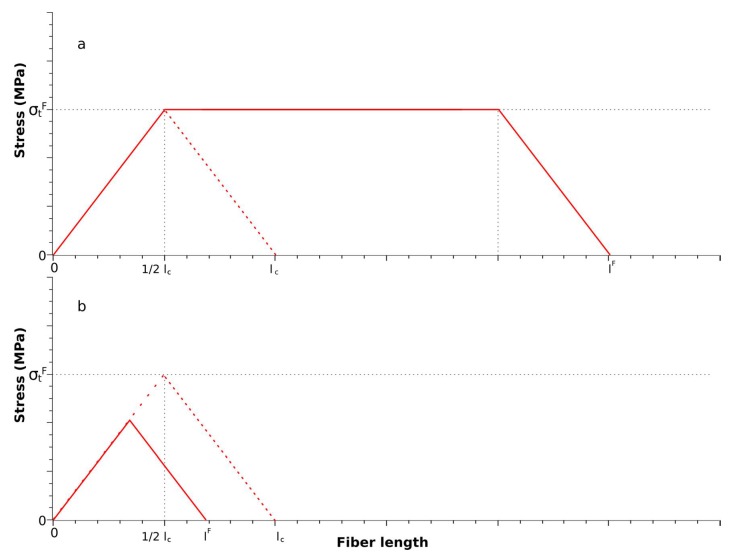
Axial load diagrams for (**a**) supercritical and (**b**) subcritical length fibers.

Kelly and Tyson presented an evolution of the modified rule of mixtures separating the contributions of the subcritical and supercritical fibers [35]. The concept of a critical length was provided by the shear-lag model used to analyze the stress distribution in reinforcements inside a composite. This model states that the matrix transmits its load to the reinforcement in the interface via shear loads. Thus, the fibers will show a null load in its ends and a full load at its center. Depending on the length of the fiber, the load at the center will be higher or lower than its intrinsic tensile strength. The fibers with enough length to be fully loaded will be called supercritical, and shorter fibers will be called subcritical (Figure 2). 

The length required to be fully loaded is impacted by the exterior area of the fibers and the quality of the interphase, or its ability to pass the shear loads from the matrix to the reinforcement. The critical length (*l_c_*) can be computed as

(7) $$l_{c}=\frac{r^{F}\cdot \sigma_{t}^{F}}{\tau }$$

where *τ* is the interfacial shear strength that limits the shear load transfer between the reinforcement and the matrix.

The modified Kelly and Tyson model has the formulation of

(8) $$\sigma_{t}^{C}=\chi_{1}\left(\overset{l=lc}{\underset{l=0}{\sum }}\left[\frac{\tau \cdot l\cdot V_{l}^{F}}{2r^{F}}\right]+\overset{\infty }{\underset{l=lc}{\sum }}\left[\sigma_{t}^{F}\cdot V_{l}^{F}\cdot \left(1-\frac{\sigma_{t}^{F}\cdot 2r^{F}}{4\cdot \tau \cdot l}\right)\right]\right)+\left(1-V^{F}\right)\cdot \sigma_{t}^{M*}$$

The reinforcements do not have a regular size and show a distribution of lengths and diameters, as shown by the morphological analysis. Thus, for any of the length ranges, obtaining a volume fraction (*V_l_^F^*) is possible. Equation (8) adds an orientation factor (*χ*_1_) to the original Kelly and Tyson equation, formulated for aligned reinforcements [13,36,37].

In its present form, Equation (8) has three unknowns, *σ_t_^F^*, *τ,* and *χ*_1_. A method was developed by Bowyer and Bader to solve the equation [38]. 

### 3.6. Bowyer and Bader Method

**Figure 3:**
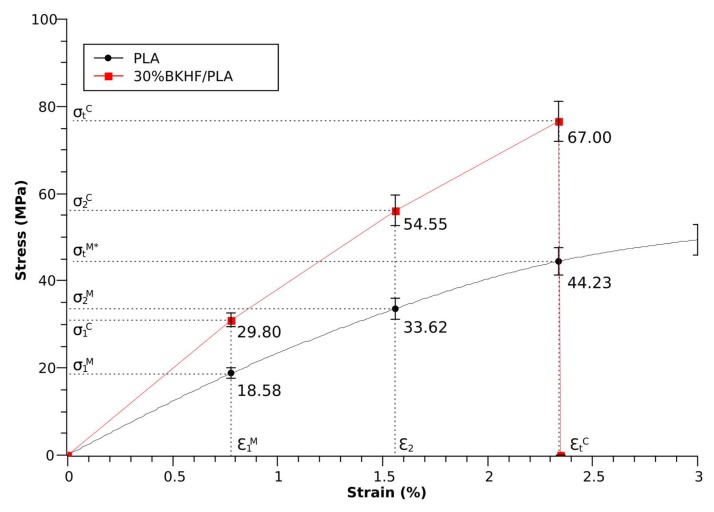
Stress-strain curves of the PLA matrix and the composite reinforced with a 30% bleached kraft hardwood fibers (BKHF). The intermediate strain points used to solve the Kelly and Tyson modified equation are indicated with the subscripts 1 and 2, respectively.

The solution proposed by Bowyer and Bader suggested changing the intrinsic tensile strength of the fiber by *ε_t_^C^ E_t_^F^*, assuming that the matrix, the composite, and the fibers will show the same strain under the same loads. This will be only true for low deformations and in the elastic zone of the composite. Bowyer and Bader proposed a solution based on collecting experimental data from two strain-stress load states between 0 and the ultimate stress of the composite (Figure 3).

Then, a numerical method was used to find the values of the interfacial shear strength and the orientation factor that solve the equation. Then, Equation (8) was used to obtain the intrinsic tensile strength of the reinforcement.

## 4. Results and Discussion

### 4.1. Bleached Kraft Hardwood Fiber Morphology

**Figure 4:**
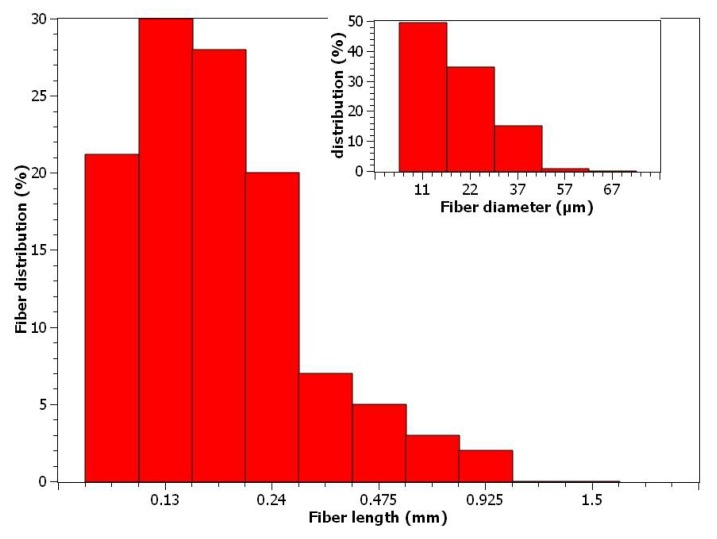
Distribution of the lengths and diameters of the BKHF, extracted from a composite with a 30% *w*/*w* reinforcement content.

The morphology of the fibers, including length and width, changes during the preparation of composite materials [39]. The length of the fibers decreases under attrition during the mixing and injection processes. The decrease in the reinforcement mean lengths are higher when the fiber content is increased, due to the higher viscosity of the composite materials, implying that more energy is required to perform a correct mixing [37]. However, the width of the fibers can change drastically due to the collapse of their lumen [40,41]. Therefore, the real impact of the morphology of the fibers on the properties of a composite must be evaluated from the fibers extracted from these composites. Figure 4 shows the distribution of the fiber’s lengths and diameters. 

The recorded mean arithmetic length and diameter of the BKSF fibers were 191.0 and 18.7 µm, respectively. Thus, the aspect ratio of the reinforcement (*l_a_*/*d^f^*) was 10.21. It is accepted that reinforcements with aspect ratios higher than 10 have good strengthening and stiffening capabilities. Figure 4 shows a high content of short fibers, whereas fewer longer fibers are present. Thus, the distribution is far from normal. Consequently, the weight and double weight lengths were computed as 336.5 and 453.7 µm, respectively. These weighted lengths were used later during the micromechanics analysis.

### 4.2. Tensile Properties of the Composites

Table 1 shows the means of the tensile strength (***σ_t_^C^***), strain at break (***ε_t_^C^***), and Young’s moduli (***E_t_^C^***) of the BKHF-based composites. The table also shows the contribution of the matrix to the tensile strength of the composite (σ_t_^M^*), evaluated as the tensile strength of the matrix at the ultimate strain of the composite.

The tensile strength of the composites evolved linearly against the reinforcement content with a correlation coefficient (*R*^2^) of 0.9934. Usually a linear behavior implies a good dispersion of the reinforcement and the presence of a medium or strong interphase between the reinforcement and the matrix. The tensile strengths of the composites with a 10% to 30% BKHF contents were 10.5%, 38.6%, and 54.3% higher than the PLA matrix, respectively. These percentages were lower than those obtained when a polypropylene matrix was used for natural fiber-based composites [13,42]. Nonetheless, the nominal values of the PLA-based composites were higher, mainly due to the initial tensile strength of. PLA with a tensile strength of 49.6 ± 1.54 MPa. PP matrixes previously used by the researchers showed tensile strengths around 28 MPa [42]. Thus, PLA’s tensile strength is 43% higher than that of PP. However, the interphase of the PP-based composites was optimized with the use of coupling agents. Compared to commodity composites like glass fiber-reinforced PP, the tensile strength of such composites is similar to those that are PLA-based [12,43]. Thus, from a tensile strength point of view, BKHF-reinforced PLA composites can replace glass fiber-reinforced PP composites.

The Young’s modulus of the composites behaved similarly to the tensile strength, evolving and increasing linearly with the reinforcement content, with a *R*^2^ of 0.9981. It is accepted that the Young’s modulus is barely impacted by the quality of the interphase. Thus, a linear behavior against the reinforcement content indicates a good dispersion of the reinforcement. The Young’s modulus of the composites with 10%, 20%, and 30% BKHF contents were 29.4%, 67.6%, and 100.0% higher than those of PLA, respectively. Like the tensile strength, the percentage values obtained with PP-based composites were higher [44,45]. Regardless, the nominal values of the PLA-based composites were still higher than those of the PP-based composites at the same reinforcement ratio. The reason for this result is that the initial Young’s modulus of the PLA is more than double that of PP (1.5 GPa) [45]. Compared with glass fiber-reinforced PP composites, the Young’s moduli of the PLA-based composites were higher at the same reinforcement content [24,45]. Thus, from a stiffness point of view, the PLA-based composites are also an alternative to glass fiber-reinforced PP composites.

PLA-based composites also showed comparatively good strains at break. PP-based composites usually show high decreases [32,42]. 

The study of the tensile properties of BKHF-reinforced PLA composites revealed materials with similar or better properties than those of commodity materials that are actually being commercialized and used in areas like automotive, product design, or as building materials. 

PP-based composites use coupling agents to obtain good interphases due to its hydrophobic nature in front of hydrophilic reinforcements [46,47,48]. The literature shows that uncoupled composites tend to maintain or decrease in tensile strength when the reinforcement content increases. Unlike these composites, uncoupled PLA-based composites showed linear increases in their tensile strength against the reinforcement contents. Thus, a compatibility exists between PLA and natural fibers. The interphase is probably composed of hydrogen bonds or van der Waals interactions between the fiber surface cellulose and holocelluloses and the PLA [43].

However, the interphase between poly(lactic acid) and natural fibers is still controversial. On the one hand, several authors demonstrated that lignin could hinder the interaction between natural fibers and poly(lactic acid) [6]. However, proper reinforcement dispersion within the matrix becomes a challenge if the hydrophilic characteristic of the fibers is too high, directly affecting the final mechanical properties of the composites. On the other hand, other studies demonstrated that moderate amounts of lignin could promote the interaction between both phases [49]. In this work, bleached kraft eucalyptus fibers were used; the surface is mainly composed of hydroxyl groups. For this reason, mimicking the strategy adopted by Granda et al., diglyme was used as the dispersant [11].

### 4.3. Net Contribution of the Fibers to the Tensile Properties of the Composite

A possible measure of the quality of the interphase is the evaluation of the net contribution of the reinforcements to the tensile strength and the Young’s modulus of the composite. The literature shows that this contribution is considerably impacted by the nature of the matrix [50,51]. Similar contributions were obtained for the same reinforcement used with different polyolefin like PP or high density polyethylene (HDPE), but when this reinforcement was used with another polymer chemical family, the contributions changed drastically [43,51]. 

**Figure 5:**
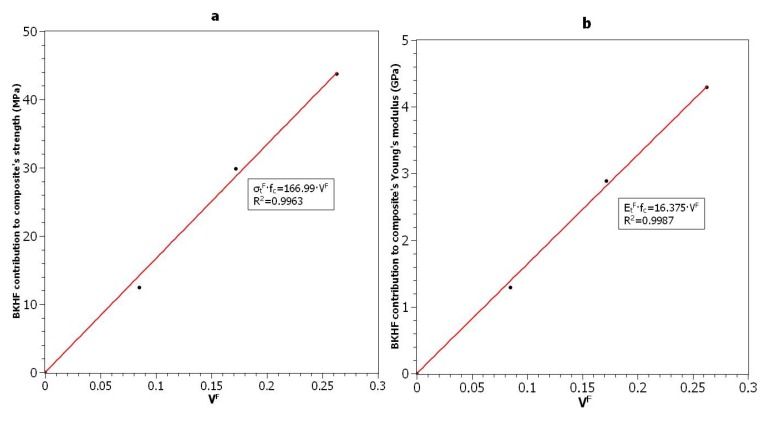
Net contributions of the reinforcement to the (**a**) tensile strength of the composite and (**b**) Young’s modulus of the composite.

The contributions of BKHF to the tensile strength and the Young’s modulus of the matrix were evaluated by means of a fiber tensile strength factor (FTSF) and a fiber tensile modulus factor (FTMF), respectively. Both factors were obtained by rearranging the corresponding modified rule of mixtures and isolating the contribution of the fibers [37,43]. Then, the factors were the slope of the linear regression curve passing through the origin (Figure 5).

When used as reinforcement for PLA, the BKHF had a FTSF value of 176.75 MPa. This value is clearly higher than softwood as a reinforcement for the same matrix (123.98 MPa), indicating that BKHF were able to create a better interphase than the softwood [43]. The FTSF of BKHF was also higher than that of wood fiber as a PP reinforcement, with a value of 109.4 MPa. Regardless, the value was also clearly inferior to that of glass fiber as a PP reinforcement with 273.85 and 427 MPa for the uncoupled and coupled composites, respectively. The neat contribution of the reinforcement indicates the presence of a strong interface or a high intrinsic tensile strength.

The FTMF revealed similar conclusions, at 16.375 MPa, it was higher than that of a natural fibers as PP reinforcement (10.87 MPa), but lower than glass fiber as a PP reinforcement (32.6 MPa) [41]. As mentioned above, the quality of the interphase has little impact on the Young’s modulus of a short fiber reinforced composite. Thus, the relatively high FTMF indicates a high value for the efficiency factor or BKHF’s high intrinsic Young’s modulus.

With the objective of finding the intrinsic tensile properties of BKHF and the quality of the interphase, a micromechanics analysis was proposed.

### 4.4. Micromechanics

#### 4.4.1. Micromechanics of the Young’s Modulus

The first step involved computing the intrinsic tensile strength of the fibers using Hirsch’s model (Equation (2)). The results are shown in Table 2.

The fibers showed a mean intrinsic Young’s modulus of 28.8 ± 1.661 GPa. The value is higher than that obtained for a bleached kraft softwood fiber, with a value of 21.2 GPa, showing the advantage in terms of the stiffening capabilities of the hardwood over softwood. Other reinforcements provided by softwood, like stone groundwood (SGW), also showed lesser intrinsic tensile strengths (18.2 GPa) [12]. Conversely, other lignocellulosic fibers, like hemp strands, were reported to have similar intrinsic Young’s modulus, with a 26.8 GPa [45]. As hemp strands can be considered high quality lignocellulosic reinforcements, the values obtained with BKHF are notable. Regardless, compared with commodity reinforcements like glass fibers that have a 71.6 GPa modulus, BKHF is clearly inferior [44]. Notwithstanding, the relative Young’s moduli, which is the ratio between the reinforcement modulus and its density, reduces the differences.

The mean efficiency factor of 0.541 was in line with those obtained for other lignocellulosic reinforcements. However, SGW-based composites were reported to have slightly higher values and hemp strand-based composites to have lower values [44]. 

The length efficiency factors showed a mean value of 0.92, very similar to those obtained for other lignocellulosic reinforced composites [44,45]. The obtained length efficiency factors were noticeably higher than the orientation factors, revealing the greater impact of the morphology of the reinforcements than its relative orientation on the Young’s modulus of the composites.

Two scientific studies completed by Fukuda and Kawada [52] and Sanomura and Kawamura [53] linked the orientation factor with a theoretical mean orientation angle of the fibers (α*_o_*). The rectangular distribution (square packing) has already been reported as adequate for short fiber semi-aligned reinforced composites [28,41,43,54]; its equation is

(9) $$\eta_{l}=\frac{\sin\left(\alpha_{o}\right)}{\alpha_{o}}\cdot \left(\frac{3-\upsilon }{4}\cdot \frac{\sin\left(\alpha_{o}\right)}{\alpha_{o}}+\frac{1+\upsilon }{4}\cdot \frac{\sin\left(3\alpha_{o}\right)}{3\alpha_{o}}\right)$$

The obtained mean orientation angle was 47.3°, very similar to other lignocellulosic-reinforced composites, despite the matrix.

The good Young’s modulus of the composites can be explained by the notable intrinsic Young’s modulus of BKHF and the high Young’s modulus of PLA. The other parameters were very similar to those of other composites.

#### 4.4.2. Micromechanics of the Tensile Strength

A micromechanics analysis of the tensile strength was performed to support the conclusions of the Young’s modulus study, focusing on the important role of the intrinsic properties of the reinforcement instead of the other parameters (morphology and orientation). The analysis of the tensile strength allowed obtaining a measurement of the quality of the interphase.

The modified Kelly and Tyson equation (Equation (7)), with the solution provided by Bowyer and Bader, were used to obtain the intrinsic tensile strength of BKHF. The study was performed for the composites with a 30% BKHF content, as the fiber extraction and morphological analysis was completed on these composites (Figure 4). The literature reports that the morphology of the fibers changes with the reinforcement content, and the composites with higher reinforcement content had shorter mean fiber lengths [36,55]. The intermediate points used to solve the equation were one-third and two-thirds of the ultimate strain. Figure 3 provides the numerical values.

The intrinsic tensile strength of BKHF was calculated as 768.4 MPa. The value was notably higher than other lignocellulosic or wood fibers, like hemp strands or stone groundwood [12,34]. Thus, as expected, the intrinsic property of the reinforcement had an important role in the tensile strength of the composite. Notably, this value was also inferior to that of glass fiber, as expected when comparing the FTSF of both reinforcements. We also found that the orientation factor was inside the expected parameters, with a value of 0.284. It is accepted that the orientation of the fibers in a mold-injected specimen is mainly impacted by the geometry of the mold and the equipment used to mold the specimen [37]. Past analyses showed that with the equipment at our labs, the orientation factor ranges from 0.25 to 0.35 [19,56]. Finally, the interfacial shear strength was evaluated at a value of 27.8 MPa. This value is very near to 28.6 MPa, the von Mises prediction for a strong interphase (*σ_t_^M^*/*3^1^*^/*2*^). Thus, the interphase between PLA and BKHF was considered as strong, in agreement with the findings of the macro-mechanical properties analysis.

The Kelly and Tyson equation allowed the comparison of the contribution of the fibers and the matrix. The reinforcements contributed 57.4% to the tensile strength of the composite, showing its high strengthening capabilities, already demonstrated during the FTSF analysis.

The modified rule of mixtures (Equation (6)) was used to compute the value of the coupling factor (Table 3). 

Composites with strong to optimal interphases have coupling factors around 0.2 [13]. All the composites showed this or slightly higher values. Thus, the hypothesis about a strong interphase between PLA and BKHF was doubly confirmed by the values of the interfacial shear strength and the coupling factor. The morphology and the interphase quality impacting the composite more than the orientation of the fibers was supported by the higher values of the length and interphase factor compared to the orientation factor.

Finally, the literature supports a relation between the orientation factor and a theoretical mean fiber orientation [13]

(10) $$\chi_{1}=cos^{4}\left(\alpha \right)$$

The resulting theoretical orientation angle was 61°. The discrepancies between the theoretical orientation angles preview by the Young’s modulus (Table 2) and the tensile strength analysis (Table 3) are worth nothing. These discrepancies have been already reported in the literature [13]. The establishment of the mean orientation angle in three dimensions is a difficult and subjective task. The mathematical function that predicts the fiber orientation factor in the modified rule of mixtures appears to be different from the mathematical function that predicts the orientation factor ($\eta_{o}$). 

## 5. Conclusions

Bleached kraft hardwood fibers from eucalyptus were used to formulate, prepare, and test PLA-based composites. These composites, with reinforcement contents ranging from 10% to 30% *w/w* showed noticeably good tensile strengths, in line with glass fiber-reinforced polypropylene composites. Compared with other lignocellulosic or wood fiber-reinforced polypropylene composites, the BKHF/PLA composites always demonstrated better tensile strength and Young’s modulus at the same reinforcement content. Thus, obtaining presumably biodegradable composites with mechanical properties comparable to commercially available non-biodegradable composites is possible.

The BKHF-reinforced PLA composites had a good interphase without using any further coupling agent or chemical treatment. This places these composites under the umbrella of green chemistry. However, further research is still needed to assess the environmental impact of such composites and the advantages compared to recycling.

The micromechanics analysis revealed the high intrinsic tensile strength and Young’s modulus of BKHF. These properties were higher than other wood fibers and comparable to high quality lignocellulosic reinforcements such as hemp strands. The analysis also revealed the higher importance of the morphology of BKHF and the quality of the interphase compared to its orientation to obtain good composite tensile properties.

## Figures and Tables

**Table 1:** Tensile strength, strain at break, and Young’s modulus of the composite. Contribution of the matrix to the tensile strength of the composite.

BKHF (%)	*V^F^*	*σ_t_^C^* (MPa)	*ε_t_^C^* (MPa)	*E_t_^C^* (GPa)	σ_t_^M^* (MPa)
0	-	49.6 ± 0.23	3.3 ^+^ ± 0.18	3.4 ± 0.11	-
10	0.085	57.3 ± 0.48	2.9 ± 0.12	4.4 ± 0.18	48.4
20	0.172	68.7 ± 1.08	2.6 ± 0.15	5.7 ± 0.22	46.2
30	0.263	76.5 ± 1.31	2.3 ± 0.07	6.8 ± 0.26	44.2

^+^ The strain of the PLA matrix was measured at its maximum strength.

**Table 2:** Intrinsic Young’s modulus of the bleached kraft hardwood fibers (BKHF) and micromechanics parameters for a modified rule of mixtures of the Young’s modulus of the composites

BKHF (%)	*E_t_^F^* (Gpa)	*η_e_*	*η_l_*	*η_o_*	*α*
10	28.09	0.541	0.905	0.598	46.6º
20	31.41	0.533	0.917	0.581	47.9º
30	29.84	0.547	0.931	0.587	47.5º
Mean	28.78	0.541	0.918	0.589	47.3
S.D.	1.661	0.007	0.013	0.008	0.66

**Table 3:** Intrinsic tensile strength of the BKHF and micromechanics parameters for a modified rule of mixtures of the tensile strength of the composites.

BKHF (%)	*σ_t_^F^* (Mpa)	*f_c_*	*χ*_1_	*χ*_2_	*α*
10	768	0.20	0.284	0.704	61°
20	768	0.23	0.284	0.810	61°
30	768	0.22	0.284	0.774	61°
Mean	768	0.22	0.284	0.763	61°
S.D.	-	0.015	-	0.054	-
